# Effect of Amber (595 nm) Light Supplemented with Narrow Blue (430 nm) Light on Tomato Biomass

**DOI:** 10.3390/plants12132457

**Published:** 2023-06-27

**Authors:** Bo-Sen Wu, Mahnaz Mansoori, Keli Trumpler, Philip Wiredu Addo, Sarah MacPherson, Mark Lefsrud

**Affiliations:** Bioresource Engineering Department, McGill University, Macdonald Campus, Sainte-Anne-de-Bellevue, QC H9X 3V9, Canada; bo-sen.wu@mail.mcgill.ca (B.-S.W.); mahnaz.mansoori@mail.mcgill.ca (M.M.); keli.trumpler@mail.mcgill.ca (K.T.); philip.addo@mail.mcgill.ca (P.W.A.); sarahanne.macpherson@mcgill.ca (S.M.)

**Keywords:** biomass, chlorophyll, greenhouse, LEDs, photosynthesis, *Solanum lycopersicum*

## Abstract

Full-spectrum light-emitting diodes (LEDs) mainly comprising 460-nm + 595-nm light are becoming a mainstay in the horticulture industry, and recent studies indicate that plant productivity under white LEDs is higher than combined blue and red LED lighting. Different light properties (wavelength and bandwidth) in full-spectrum light, particularly for the blue and amber light regions, have only partly been explored. This research aimed to characterize the effects of amber + blue light wavelengths and bandwidths on tomato (*Solanum lycopersicum* cv. Beefsteak) growth, morphology, and production efficiency. Tomato seedlings were subjected to four different light treatments for 60 days: narrow amber light (595 nm), narrow blue + narrow amber light (430 nm + 595 nm) with a 1:10 ratio, white LED (455 nm + 595 nm), and a high-pressure sodium (HPS) lamp (control). The highest mean fresh mass yield occurred with the narrow blue + narrow amber light (479 g), followed by white LED at 20% less, HPS at 34% less, and narrow amber at 40% less. Dry mass and plant height were similar among light treatments. Supplementing narrow amber light with 430-nm blue light led to a 20% increase in chlorophyll content. Findings indicate that narrow amber light is more efficient in biomass accumulation than broad amber light and that precise selection of different blue and amber wavelengths can greatly impact the growth and development of tomato seedlings. This energy-efficient narrow-wavelength combination shows improvement over white LED lighting for maximizing tomato growth.

## 1. Introduction

Light-emitting diodes (LED) have become common electrical lighting systems in controlled environment agriculture (CEA). LEDs have a high energy conversion efficiency (>50%) and offer better control over intensity and spectral emission [1,2]. Because of these technical features, LEDs are now widely used in plant photobiology research [3,4]. Massa et al. (2006, 2008) highlighted the importance of lighting on plant development on earth and in space. Narrow-spectrum LEDs can be used to manipulate plant morphology and physiology in a way that is unachievable with conventional lighting sources [5,6]. For example, combined blue and red LED lighting can improve seedling quality and growth [7] and protect plants from diseases [8], in addition to controlling stem elongation and flowering [9]. Various research studies using tomatoes show enhanced biomass and fruit yield under a mixture of blue and red LED treatments when compared to high-pressure sodium (HPS) [10,11,12].

Many questions remain around LED light emitted within the visible spectrum. Most studies are conducted with blue and red LEDs, likely because these two LED wavelength regions are reportedly more energy-efficient in CEA production [13]. However, they are not the most efficient light wavelengths at driving photosynthesis. Although McCree (1972) reported the highest quantum yields for more than 25 plant species using red light (625 nm), these data were not significantly different (*p* < 0.05) from plants treated with amber light (575 nm). Studies later confirmed that amber light augments dry mass accumulation [14], while supplemental amber light results in better plant productivity and development [15,16]. One recent study demonstrated that broad-spectrum 595-nm light yielded 50% higher fresh and dry mass than other red LEDs commonly used in CEA [17]. White LEDs comprised of amber-rich lighting likewise showed better photosynthetic activity than a blue and red light mixture [18]. Due to lighting technology limitations, these studies were conducted with varying bandwidths of amber light. For example, a 10-nm bandwidth from a low-pressure sodium lamp [14], a 25-nm bandwidth from monochromator-filtered light [13], and a 70–80-nm bandwidth with full-spectrum LEDs [15,17] have been reported. This large variation in the amber light spectrum creates challenges when evaluating the impact of amber light on plant production.

Isolating monochromatic light is a widespread approach used to study the effect of light wavelength on photosynthesis, although it can lead to a lower quantum yield compared to dichromatic or full-spectrum light [19]. Blue light is often used to supplement wavelengths, as it is essential for plant growth and development [20,21]. Currently, 450–460 nm light is the most common blue wavelength range used in plant LED lighting research. This is because of its proven ability to induce high plant productivity when combined with red light [22] and its high energy conversion efficiency [23]. It is the basic LED component used to produce “white” LED light, and most of our understanding of blue light and plant growth to date has focused on this 450–460 nm region. The light-emitting mechanism of white LED light has limited further exploration of this important spectral region when investigating full-spectrum light. Current lighting technology adds further constraints to the end user’s selection of only blue wavelengths of 450–460 nm light.

Studies on green and amber light are not as extensive as those examining blue and red light due to theories that consider them inessential. Research studies show that 43–87% of green light can be absorbed by the plant and efficiently used for photosynthesis [24,25]. Studies have shown that amber light causes an increase in photosynthetic action [13,26] while exhibiting suppressed plant growth as a plant stress response under high and saturating light intensities [27,28]. Inversely, lower light intensity results in higher plant productivity [29,30]. This narrow-spectrum light may be bypassing the photoreceptor sensing responsible for the structural developmental processes of the plant. Broad-spectrum amber light containing red and far-red wavelengths is triggering phytochromes as a high irradiance response and shade avoidance mechanism [31,32].

The objective of this study was to investigate and evaluate the impact of light spectra and bandwidths, particularly narrow blue wavelengths and different amber light bandwidths, on tomato plant growth. Tomato seedlings were cultivated under four light treatments, including three different LED spectra and an HPS spectrum, in a controlled environment. Results from this study provide new data that may help lighting manufacturers and CEA growers determine essential wavelengths for tomato growth.

## 2. Results and Discussion

Light quality and quantity are paramount to plant cultivation. Photomorphogenesis refers to plant growth parameters affected by light and regulated by plant light receptors [33]. In this study, the effects of combinatory blue and amber light with different bandwidths on plant growth were explored. Tomato seedlings were cultivated under four different light treatments for 60 days to compare biomass production and morphology. Light treatments were narrow amber (595 nm) light, narrow blue (430 nm) + narrow amber (595 nm) light, white LED (blue, 455 nm + broad amber, 595-nm) light, and HPS (control). Average PPFD of 213.4 ± 13.1 µmol m^−2^ s^−1^, 241.8 ± 32.3 µmol m^−2^ s^−1^, 283.4 ± 46 µmol m^−2^ s^−1^ and 236 ± 39.5 µmol m^−2^ s^−1^ were recorded for narrow amber, narrow blue + narrow amber, white LED, and HPS, respectively.

### 2.1. Plant Growth (Fresh Mass and Dry Mass)

Fresh and dry mass data for tomato plants grown under the different experimental light treatments for 60 days are compared in Table 1. Although plant growth response data was comparable among light treatments, the narrow blue + narrow amber light treatments promoted the highest fresh mass (FM), followed by white LED, HPS, and narrow amber light treatments. Compared to the HPS control, narrow amber light reduced FM by 9.9%, while narrow blue + narrow amber and white LEDs increased FM by 34 and 17.2%, respectively. No significant (*p* < 0.05) difference was recorded for FM from plants treated with the narrow blue + narrow amber, and white LED treatments. No significant difference (*p* < 0.05) in dry mass (DM) was observed between light treatments. The lowest biomass (FM and DM) observed was with narrow amber light; this was expected since it has been demonstrated that 100% monochromatic light results in lower DM than full-spectrum light [28,34]. Previous studies have shown that low-pressure sodium lamp light, which has a similar light spectrum to narrow amber light, resulted in a 15% decrease in DM in soybean (*Glycine max*), sorghum (*Sorghum bicolor*) [28], and potato (*Solarium tuberosum*) when compared to fluorescent lamps [34].

Supplementing narrow amber with narrow blue light in this study resulted in a 39.9% increase in FM compared to the monochromatic narrow amber light treatment. It can be hypothesized that the addition of blue light is necessary for tomato plant development and that of other plants such as cucumber and cannabis [35,36]. Blue light triggers stomatal aperture opening driven by phototropins (Phot) 1 and 2 and, therefore, access to CO_2_, ultimately enhancing photosynthesis [37,38,39]. Hsiao et al. (1973) and Sharkey and Raschke (1981) showed a stomatal action spectrum with a peak between 420 and 460 nm in the blue light region of *Vicia faba* and *Xanthium strumarium*, respectively. Additionally, blue light inhibits hypocotyl elongation and promotes biomass production [40], triggers phototropism, leaf thickness, chlorophyll content [37], stem elongation, and leaf expansion; however, high blue light intensity can inhibit plant growth [41].

White LED light, comprised of 455 nm and broad amber (595 nm) light, is slowly becoming the standard lighting in CEA [2,20]. This light mixture results in improved photosynthesis and plant growth compared to the combined blue and red LED light, particularly with the low color temperature of white LEDs that emit a lower blue light percentage [15,18]. Shifting the supplemental blue light wavelength from 455 nm to 430 nm and narrowing the amber spectrum in this study resulted in a 25% DM increase. The rationale for moving the maximum wavelength from 455 nm to 430 nm was for maximum photosynthetic efficiency in the blue region and to limit the chloroplast movement mechanism that occurs primarily at 450 nm [42].

Although higher quantum yields have been reported in different crops using 430 nm light compared to 450–460 nm light [13,26], this high quantum yield does not appear to lead to high biomass accumulation [43]. Different supplemental blue wavelengths, including 430 nm and 460 nm light, on pak-choi (*Brassica rapa*) were compared for growth, and comparable FM and DM were obtained. Between these two light treatments, in this work, it is difficult to conclude if the increased FM was caused by shifting the blue light wavelength or narrowing the amber spectrum. It may be that the observed increase in both FM and DM under narrow blue + narrow amber treatment is partially caused by different bandwidths in amber light and that narrow amber light is more efficient at inducing biomass accumulation than broad spectrum amber light. Further investigation with proper spectrum design and newer LED technology could help better understand the impact of these two specific wavelengths.

The limited literature available for amber light and plant growth shows conflicting data [14,27,44]. Dougher and Bugbee (2001) reported that an increased fraction of amber light (580–600 nm) provided with single-ended HPS lamps suppressed lettuce (*Lactuca sativa*) growth at two PPFD levels (200 and 500 µmol m^−2^ s^−1^). Although the regression data for DM showed a linear decrease, the raw data from this study indicated a quadratic response, where the peak corresponds to an amber light fraction of ~25%. Furthermore, a small fraction of blue light, peaking at 460 nm and 510 nm, existed in this experimental spectrum. Increased blue light resulted in suppression of plant growth (FM and DM) [45,46], and this effect would carry on while increasing the PPFD levels of HPS lamps. Therefore, this effect cannot be determined using “broad-spectrum” HPS lighting. It is possible that the reported suppression effect on lettuce growth is caused by 460 nm and 510 nm light from HPS lighting, and this may explain why shifting the blue wavelength from 430 nm (430 + 595 nm treatment) to 500 nm (HPS) led to a decrease in both FM and DM in this study.

### 2.2. Chlorophyll (Chl) Content, Height, Stem Diameter, and Number of Internodes

**Figure 1:**
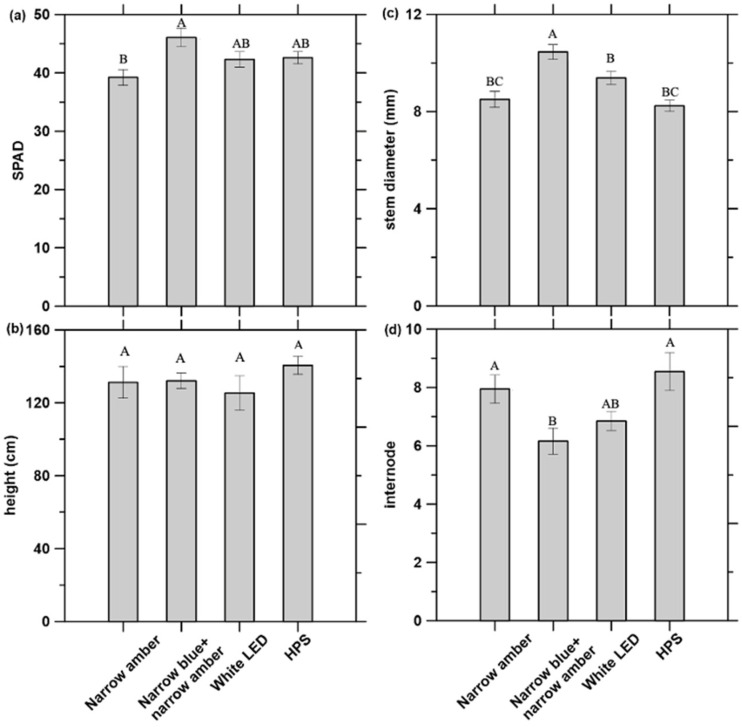
Effect of the different experimental light treatments on tomato plant growth (*n* = 6): (**a**) chlorophyll content (SPAD values), (**b**) height (cm), (**c**) stem diameter (mm), and (**d**) number of internodes. Bars with different letters represent significant differences using Tukey’s post hoc test. LED: light emitting diode; HPS: high-pressure sodium.

The impact of the four different experimental light treatments used on chlorophyll (Chl) content, height, stem diameter, and number of internodes of tomatoes is presented in Figure 1.

#### 2.2.1. Chlorophyll (Chl) Content

**Figure 2:**
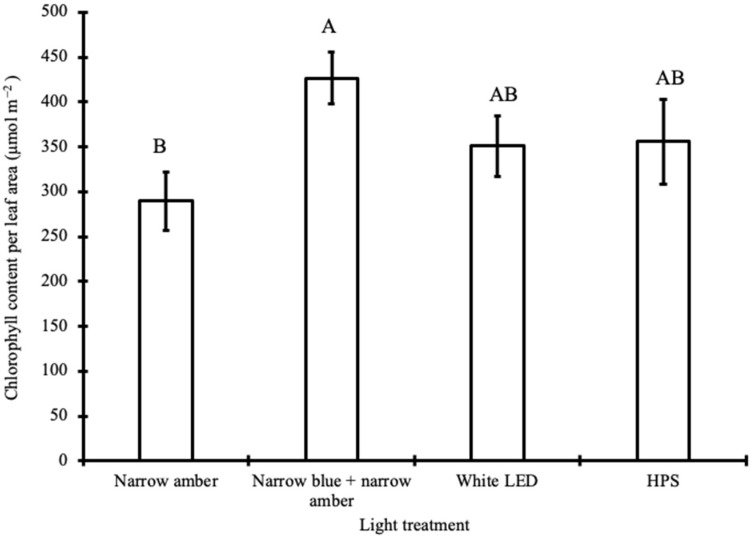
Chlorophyll content per leaf area (µmol m^−2^) measured for tomato plants under the different light treatments. Bars with different letters represent significant differences using Tukey’s post hoc test. LED: light-emitting diode; HPS: high-pressure sodium.

Chl content was similar among treatments, except for the narrow amber and narrow blue + narrow amber light. The highest Chl content (SPAD values) in the tomato plant was observed under narrow blue + narrow amber light (46.07 ± 1.58), followed by HPS (42.59 ± 1.04), white LED light (42.31 ± 1.31), and narrow amber light (39.23 ± 1.32) (Figure 1a). Total Chl content is associated with light quality [47], and 450–460 nm light promotes Chl accumulation [48,49]. The data collected in this study with narrow blue + narrow amber light are only in partial agreement with these findings. Narrow blue + narrow amber light led to a 20% increase in Chl content compared to the narrow amber light treatment. No significant (*p* < 0.05) difference in Chl content was observed when white LED light and HPS were compared. Total lower Chl content in *Arabidopsis thaliana* grown under amber light has been reported when compared with other monochromatic light in the PAR spectrum [44]. Although our data does not explain the interaction effect between narrow amber and narrow blue light on Chl accumulation, it is plausible that this wavelength combination suppresses Chl biosynthesis, and this merits further examination. Xiong et al. (2015) investigated the relationships between SPAD and chlorophyll content per leaf area for monocots and dicots. SPAD values are directly correlated (r^2^ = 0.9) with the Chl content per leaf area of tomatoes [50]. Although destructive Chl analyses were not performed for this study, the model (Equation (1)) generated by Xiong et al. (2015) was used to obtain the Chl content per leaf area for the tomato plants under different light treatments. Data presented in Figure 2 demonstrate that plants treated under narrow blue + narrow amber light have the highest Chl content per leaf area. Statistical analyses did not show significant (*p* < 0.05) differences between tomato plants treated under white LED light and HPS.

SPAD = (0.0492 × chlorophyll content per leaf area) + 25.084(1)

#### 2.2.2. Height, Stem Diameter, and Number of Internodes

Tomato plants grown for 60 days under HPS (control) showed the highest plant height (140.56 ± 4.89 cm), followed by narrow blue + narrow amber (132.10 ± 4.33 cm), narrow amber light (131.22 ± 8.72 cm), and white LED light (125.39 ± 9.43 cm) (Figure 1b). Amber light, or a light spectrum with a low blue/red ratio, results in strong shoot elongation [28,51], and blue light shortens plant height [45,46]. In this study, no significant (*p* < 0.05) difference in plant height was observed between light treatments, with or without blue light (Figure 1b). Further to this, no dwarfing effects from supplementing with blue light wavelengths (430, 455, and 500 nm) were observed. The highest stem diameter measured was 10.45 ± 0.3 mm with narrow blue + narrow amber, followed by white LED light (9.38 ± 0.27 mm), narrow amber light (8.50 ± 0.33 mm), and HPS (8.23 ± 0.23 mm) (Figure 1c). HPS (8.5 nodes plant^−1^) and narrow amber (8 nodes plant^−1^) light treatments resulted in higher internode counts than under the narrow blue + narrow amber treatment (6.0 nodes plant^−1^), yet there was no significant (*p* < 0.05) difference when comparing white LED light to the other light treatments (Figure 1d).

## 3. Materials and Methods

### 3.1. Plant Materials

Experiments were conducted at McGill University’s Macdonald Campus (Sainte-Anne-de-Bellevue, QC, Canada). Tomato seeds (*Solanum lycopersicum*, OSC seeds, Waterloo, ON, Canada) were placed in presoaked rockwool cubes (25 by 25 by 30 mm, Grodan, Etobicoke, ON, Canada) in germination trays (0.28 by 0.54 m, Mondi Products, Vancouver, BC, Canada). A full-strength Hoagland nutrient solution was used after the seeds germinated [52]. Trays were placed into a growth chamber (TC30, Conviron Controlled Environment Ltd., Winnipeg, MB, Canada) for 25 days with a constant temperature of 22 ± 1 °C, 50% relative humidity, and a 16 h d^−1^ photoperiod.

### 3.2. Light Spectra

**Figure 3:**
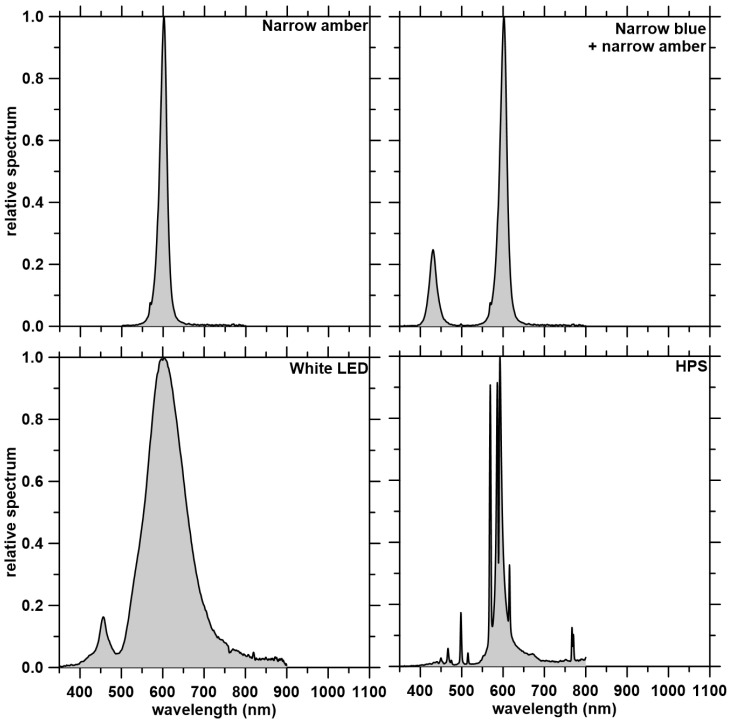
Relative spectra of the four experimental light treatments: narrow amber light, narrow blue + narrow amber light, white LED light, and high-pressure sodium (HPS) light (control).

Light treatments comprised four different light fixtures: a narrow amber light (595 nm, VQ-GLIB600W, VANQ Technology Co., Ltd., Shenzhen, China); a combined narrow blue + narrow amber light with a 1:10 ratio (430 nm + 595 nm, IL-BP700, Shenzhen Idea Light Ltd., Shenzhen, China); a white LED light (blue, 455 nm + broad amber, 595 nm, 1400 K, EcoAdvance-Slim-150W, U Technology, Calgary, AB, Canada); and a single-ended HPS lamp (ED 18 400W, HPS lamp, Philips, Amsterdam, The Netherlands). Spectra were determined with a spectroradiometer (PS-300, Apogee, Logan, UT, USA). Table 2 shows the spectral characteristics of the four experimental lighting treatments. The relative spectra of the four experimental light treatments are presented in Figure 3. The narrow blue + narrow amber, and narrow amber LED fixtures were air-cooled via fans that drew greenhouse air into the center of the LED chips, and the white LED fixture was cooled using a heat sink.

### 3.3. Greenhouse Growing Conditions

The study was conducted from Fall 2018 to Winter 2019. After the 25-day germination period in the growth chamber, six tomato seedlings were selected, transplanted into the greenhouse, and cultivated for 60 days in four ebb and flow-style recirculating hydroponic systems. These were placed on a long wire-mesh table (1.2 m high) situated in a 7.6 m × 12 m greenhouse bay (set in a North-South orientation, in the North-West corner of the greenhouse) that was divided into four sections, one for each different light treatment. The hydroponic nutrient solutions were pumped using a submersible pump (Aquakingdom SP1200 submersible pump, Guangzhou, Guangdong, China) from containers located below the growing beds. The irrigation schedule was set at 10 min every hour from 7:00 a.m. to 12:00 a.m. A total of 50 L fresh, double-strength Hoagland’s solution was provided and replaced every seven days for each container. The pH and EC were maintained at 6.26 ± 0.034 and 4.10 ± 0.11 dS m^−1^, respectively. Before and after Hoagland’s refilling each week, the pH and electrical conductivity (EC) values of each container’s nutrient solution were measured with a portable pH meter (Oakton waterproof pH tester 30, EuTech Instruments, Vernon Hills, IL, USA) and a portable EC meter (combo EC meter, Hanna Instruments, Ann Arbor, MI, USA), respectively. Two air pumps (Elite 802 and Marina 200, Rolf C. Hagen, Baie d’Urfé, QC, Canada) provided oxygenation to the nutrient solutions.

Each experimental light treatment zone was surrounded by a double layer of 2.44 m of 80% shade cloth (8MK808, Harnois, St.-Thomas, QC, Canada), hanging from the upper frame of the greenhouse. All light treatments comprised a 16-h photoperiod and an 8.64 mol m^−2^ light integral. The PPFD delivered by the four experimental lighting systems was controlled by adjusting their proximity to the top of the plant canopy weekly using a light meter (LI-250A; LI-COR Inc., Lincoln, NE, USA) with an underwater quantum sensor (LI-192, LI-COR Inc., Lincoln, NE, USA). Light mapping showed that the shade cloth prevented 96% of the light from neighboring light treatments from passing through, which reduced the light interference between treatments. This allowed for independent analyses of all sections. All lighting fixtures were suspended above the center of each growing area below the double layer shade cloth to avoid light interference from sunlight. Before starting the experiment, light mapping for photosynthetic photon flux density (PPFD) level and stray light testing were conducted, with the aim of delivering 240 µmol m^−2^ s^−1^ for each light treatment. The PPFD level at each plant location was determined at the beginning of the experimental run, at the end of the experimental run, and once in between. During cultivation, plants were randomly repositioned every three days to avoid inconsistent PPFD levels within the treatment zone. Measurements were made at each plant location and at 17–22 cm (equivalent to the experimental tomato seedling height). Stray light testing showed that light interference from neighboring light treatments was less than 4%. Statistical analyses showed no significant differences between the light intensities used for the different treatments. Using the Clear Sky Calculator (https://www.clearskycalculator.com, accessed on 8 June 2023), estimated maximum PPFD for sunlight was 1481 µmol m^−2^ s^−1^ for the study’s duration. Based on the light mapping data collected before, during, and after the study, maximum sunlight interference was <8%. Temperatures in the greenhouse were set at 20 ± 1 °C during the day and 17 ± 1 °C during the night. A sensor monitoring temperature and relative humidity (S-THB-M002, OnSet, Hobo, Bourne, MA, USA) was placed at approximately mid-canopy level for mature plants in each compartment, and data were recorded every 10 min during the entire experiment.

### 3.4. Plant Growth Parameters and Measurements

After 60 days in the greenhouse, tomato plant fresh mass (FM), dry mass (DM), stem diameter, chlorophyll content (Chl), plant height, and number of internodes were recorded. FM and DM were measured using a balance (APX-153, Denver Instruments, Bohemia, NY, USA). Plants were dried at 40 °C (s.d. ± 1 °C) for 4 d prior to measuring dry mass. To verify dryness, plants were weighed every 24 h to ensure a <5% change. Stem diameter was measured at the internode above the cotyledons using a vernier caliper (Mitutoyo 530-316, Mitutoyo Canada Inc., Saint-Laurent, QC, Canada). Plant height was measured from the main stem base to the top of the young plant tissue. Chlorophyll content was measured using fresh attached leaves with the SPAD meter (Spectrum Technologies, Chlorophyll Meter SPAD-502Plus, Konica Minolta, Sakai, Osaka, Japan).

### 3.5. Statistical Analysis

Each light treatment with six seedlings was replicated three times. The data represent the mean values of the three replicates with their standard error (S.E.). RStudio version 1.1.383 (RStudio, Inc., Boston, MA, USA) was used for analysis of variance (ANOVA) with a significance level of 0.05 between the different treatments. A post hoc test between treatment comparisons was done using the Tukey’s honest significant difference (HSD) test. The given *p*-values were calculated by ANOVA to test the differences in parameters between the wavelength treatments.

## 4. Conclusions

The light spectrum considerably impacts growth development in tomato plants. The data reported here supports the body of literature that describes the different effects of light quantity (intensity) and light quality (wavelength) on plant growth and plant pigment accumulation. Narrow amber light supplemented with narrow blue light yields greater biomass than monochromatic narrow amber light, regardless of bandwidth. Data highlight the importance of optimizing light wavelengths for maximizing plant production, and consideration for light wavelengths other than the commonly used blue or red wavelengths is recommended. Future studies could expand on this research by deploying light qualities richer in the 430 nm and 595 nm wavelength regions of the spectrum while modifying their ratios and intensity levels to optimize tomato plant production.

## Figures and Tables

**Table 1:** Fresh and dry mass of tomato plants grown under different light treatments for 60 days (*n* = 6).

Light Treatments	Fresh Mass (g)	Dry Mass (g)
Narrow amber	287.79 ± 37.08 ^b^	20.09 ± 2.76 ^c^
Narrow blue + narrow amber	479.00 ± 28.28 ^a^	28.53 ± 2.30 ^c^
White LED	382.20 ± 30.70 ^ab^	26.41 ± 2.36 ^c^
HPS (control)	316.16 ± 22.71 ^b^	21.79 ± 2.19 ^c^

Values in the same column with different letters represent a significant difference using Tukey’s post hoc test.

**Table 2:** Spectral characteristics of the four experimental lighting treatments.

	Light Treatment			
	Narrow Amber	Narrow Blue + Narrow Amber	White LED	HPS (Control)
Relative single-band photon flux density (%)				
400–450 nm	0.00	19.00	1.89	1.65
451–500 nm	0.00	1.78	3.43	4.17
501–550 nm	0.89	0.70	9.57	2.16
551–600 nm	50.26	39.82	31.36	63.02
601–650 nm	47.67	37.77	34.21	20.84
651–700 nm	1.18	0.93	14.67	5.41
701–750 nm	0.00	0.00	1.89	1.65

## Data Availability

Not applicable.
